# Feasibility and acceptability of a peer-led mindful self-compassion program for adults with eating disorder symptoms

**DOI:** 10.1186/s12888-025-07325-x

**Published:** 2025-09-26

**Authors:** Maren Kjeldstad, Ingunn Mundal, Irene Kingswick, Håvard Horndalen Tveit, Mariela L. Lara-Cabrera

**Affiliations:** 1 Norwegian Resource Center for Eating Disorders (ROS), Trondheim, Norway; 2 Regional Centre for Child and Youth Mental Health and Child Welfare, Department of Mental Health, Faculty of Medicine and Health Sciences, Norwegian University of Science and Technology, Trondheim, Norway; 3 Department of Health and Social Sciences, Molde University College, Molde, Norway; 4 Central Norway Regional Health Authority, Stjørdal, Norway; 5 Department of Mental Health, Faculty of Medicine and Health Sciences, Norwegian University of Science and Technology, NO-7491 Trondheim, Norway; 6 Nidelv Community Mental Health Centre, Division of Psychiatry, St. Olavs University Hospital, Trondheim, Norway

**Keywords:** Eating disorders (EDs), Group intervention, Internet-based program, Mindful self-compassion (MSC), Mental health, Participatory research, Patient satisfaction, Peer-led, Person-centred, User involvement, Well-being

## Abstract

**Background:**

The implementation of peer-led programs provided by individuals with lived experience of mental disorders has been increasingly acknowledged as a key component in improving the quality of mental health services. When considering research on eating disorders, peer-led online programs may be an alternative approach to enhancing participants’ well-being. Furthermore, increasing evidence suggests that mindful self-compassion (MSC) may be a helpful approach to strengthening self-compassion as a prevention program for eating disorders. However, the feasibility and acceptability of online group-based peer-led MSC programs remain unexplored in this context.

**Methods:**

In this pilot study, using a single-arm pre-post pilot design, lived experience experts delivered and managed the program themselves and collected the data. Participatory action research principles informed the study’s quantitative design. Adults with symptoms of eating disorders were invited to participate. The primary outcome was to assess the feasibility and acceptability of the MSC program, measured by the dropout rate, attendance rate, and client satisfaction. As a secondary outcome, the program’s preliminary efficacy on self-compassion and psychological well-being was investigated.

**Results:**

Twenty-five people contacted the user organization, and all agreed to participate. However, 18 participants (78%) responded to the questionnaires at both measurement points. Two participants dropped out. The attendance rate was excellent, with 35% of the participants achieving 100% attendance, 39% completing seven of the eight group sessions (87.5% attendance), and 26% of the participants achieving 75% attendance. The participants reported to be very satisfied with the MSC program, with a total mean score of 14.4 (*SD* = 1.42, 95% CI [13.7, 15.1]) out of a maximum of 16 points. The participants also showed significant improvement in self-compassion and psychological well-being.

**Conclusions:**

The findings indicate that the program was both feasible and acceptable for adults with eating disorder symptoms. Additionally, participants reported significant improvements in self-compassion and psychological well-being. However, as this was the first study to examine such a program in this context, these preliminary findings warrant further research. Nonetheless, these findings suggest that peer-led online MSC programs may offer an accessible, community-rooted, and person-centered addition to ED services, particularly for individuals underserved by traditional models.

## Background

Eating disorders (EDs) constitute a group of serious psychiatric conditions characterized by maladaptive eating behaviors and dysfunctional weight-control strategies [1]. They are associated with considerable physical health impairments and significant disruptions in psychosocial functioning [2]. Early interventions have been increasingly valued and explored given the high prevalence of food, body image, and psychological concerns [3, 4]. These interventions should ideally occur between the onset of symptoms and the first specialized treatment, to prevent or reduce illness progression. Although most research has been conducted on older adolescents and university students, prevention and early intervention programs in the context of EDs appear to significantly reduce certain risk factors, promote symptom recognition, and encourage help-seeking behavior [3].

Living with an ED can significantly affect one’s quality of life, well-being, and self-compassion. Given that EDs are often marked by high levels of shame, perfectionism, and body dissatisfaction [5], self-compassion has emerged as a particularly important factor in both the development of novel intervention approaches and the recovery process [6]. Self-compassion, referring to how people relate to themselves regarding personal suffering, inadequacy, or perceived failure, can also be understood as the approach people take to emotions and distressing thoughts that affect their mental and physical well-being [7]. Reviews suggest that individuals with EDs tend to have lower self-compassion than their healthy counterparts [8–10]. Moreover, higher levels of self-compassion are associated with reduced ED symptom severity [9]. Empirical studies have indicated that both underweight and overweight individuals tend to report lower levels of self-compassion than those of average body weight, which potentially increases their vulnerability to developing EDs [11].

Studies have also found that the barrier to practicing self-compassion is more robust in people with EDs [12, 13]. Increasing evidence has identified the extent and negative impact of weight stigma, particularly in populations with higher BMIs [14]. Self-compassion has emerged as a well-established resilience factor that may buffer the adverse psychological consequences of discrimination, mitigating psychological distress, and counteracting the negative impact of weight-related stigma [15].

Enhancing self-compassion may play a pivotal role in reducing self-criticism and alleviating symptoms associated with EDs [16], making it a valuable component of treatment programs. In response to this, mindful self-compassion (MSC) has emerged as a program designed to improve self-compassion while addressing the emotional and cognitive barriers that adults with EDs can face. Developed in 2013 by Germer and Neff, the MSC program aims to enhance psychological resilience and self-compassion and is considered a hybrid program suitable for both general and clinical populations [10]. MSC approaches integrate mindfulness and self-compassion [17], as mindfulness skills are crucial for the ability to show oneself compassion [10]. However, the MSC program spends more time explicitly teaching self-compassion skills to maximize its benefits, rather than focusing on techniques to enhance mindfulness [10]. MSC has been shown to be effective in enhancing mental, emotional and physical well-being, as well as reducing anxiety, depression, and physical pain, among other benefits [7, 18]. Programs focused on self-compassion are effective for eating pathologies, body-image challenges [10], and the enhancement of self-esteem [19]. Studies have demonstrated the feasibility of MSC group-based programs led by healthcare professionals [18, 20–22].

However, several knowledge gaps need to be addressed. Firstly, the feasibility and potential benefits of group-based MSC programs led by individuals with lived experience through role modelling and active engagement, remain largely unexplored, despite these programs being increasingly recognized as a vital component in enhancing mental health services. Adults with lived experience are playing an expanding role in both mental health services and research [23–27]. Their involvement has been documented during the design and development of programs [27–31], the delivery of services [23, 32–34], and the evaluation of peer-led programs [26, 27, 32]. While collaboration with patients and user organizations in the planning and delivery of programs has become increasingly used, individuals with lived experience are still seldom engaged as equal partners in the co-creation process [34, 35]. This highlights the need to deepen our understanding of peer-led practices in which lived experience experts take on active roles as both program leaders and research collaborators [24]. Such involvement can contribute to more effective, inclusive, and person-centred approaches for individuals experiencing eating difficulties.

Secondly, emerging evidence highlights the potential clinical benefits of digital and peer-led online programs for individuals with severe mental disorders [27, 36, 37]. Although research in this area is still in development [35, 38–40], preliminary findings suggest these approaches are promising [27, 35, 38] and gaining traction, particularly in the context of EDs [38, 39, 41]. Despite their feasibility [27, 42], most evaluations of online programs occur within research-funded settings [39]. Notably, no studies have yet examined the feasibility and acceptability of an online, group-based, peer-led program for individuals with diverse ED symptoms. Given the role of self-compassion in recovery, a structured digital intervention, such as MSC program, may help bridge this gap by combining online accessibility with peer-led support**.**

Thirdly, given the importance of evaluating service quality as a key aspect of understanding patient treatment experiences [43, 44] and promoting patient-centred care [45], it is essential to assess the feasibility of programs for individuals with diverse ED challenges. Accordingly, studies examining the acceptability of an online, peer-led version of the MSC program for individuals with ED symptoms are warranted. Thus, the primary objective of this study was to evaluate whether this program is feasible and acceptable for this population. The second objective was to evaluate the preliminary efficacy of this participatory approach and investigate how it impacts self-compassion and the psychological well-being of participants.

## Methods

### Study design

This study employed a single-arm pre-post pilot design, to assess the feasibility, acceptability, and preliminary outcomes of an online peer-led MSC program. Participatory action research principles [46] informed the study’s quantitative design.

#### Participatory Action Research (PAR)

PAR principles promote collaboration, co-learning, and action-oriented inquiry involving those directly affected by the issue under study [46]. It is also described as situational, collaborative, participatory, and self-evaluative in nature, as well as being an excellent tool for narrowing the theory–practice gap through collaboration [47]. This research was conducted in close partnership with the Norwegian Resource Centre for Eating Disorders (ROS), a user-led organization offering low-threshold services—defined as accessible, free of charge, and tailored to specific target groups, with a strong emphasis on user involvement [48].

#### Delivery

PAR values guided the delivery of the program [49]. Lived-experience experts from ROS were not only central to the delivery of the program but also played key roles in managing the study and collecting data. The course instructor, who led the program, brought both professional qualifications—including a master’s degree in mental health work — as well as personal experience with EDs. The course instructor had also completed teacher training in MSC with the program’s founders, Neff and Germer. The MSC program was conducted as part of her role as managing director of ROS, further embedding the study within a community-led context**.** Following PAR’s emphasis on valuing a balance between experiential knowledge alongside academic expertise [50], this study focused on user involvement from planning the research to delivering the program. This collaborative process between ROS staff and researchers ensured that the content was both evidence-informed and grounded in lived experience.

#### Interpretation

The interpretation of the findings was also participatory. The course instructor and the research assistant contributed to the analysis and reflection on the results based on their dual perspectives as both practitioners and peers with a lived experience of an ED. Moreover, to enhance transparency and replicability, this study adhered to the Intervention Description and Replication checklist [51] and the Guidance for Reporting Involvement of Patients and the Public [52], both of which align with the participatory ethos of PAR.

### Recruitment and participants

The ROS organization recruited the participants according to PAR’s emphasis on community engagement. The ROS advertised the MSC courses through its website and social media channels. This approach encouraged ROS members to take a more active role in the recruitment process so that recruitment was inclusive and accessible.

Individuals who expressed interest were contacted by the first author for an online meeting, during which they received information about the study. This meeting also served to assess whether participation would be appropriate and potentially beneficial for each individual. Participants were not formally diagnosed during the online meeting. Only individuals aged 18 or older, who have self-reported symptoms consistent with an ED and provided informed consent, were included in the study.

### Data collection, outcomes and measures

The data were collected through an anonymous online survey before (T1, before the program) and after course participation (T2, after the program). Table 1 lists a summary of the outcomes and the timing of assessment.

**Table 1:** Data collection and measurement points

Data collected	Measurement points	
	**T1**	**T2**
Sociodemographic data	X	X
Primary outcomes		
Feasibility outcomes (dropout and attendance rates)		X
Acceptability outcome (CSQ-4)		X
Secondary outcomes		
SCS	X	X
WHO-5	X	X

*T1* before the program, *T2* after the program, *CSQ-4* Four-item Client Satisfaction Questionnaire, *SCS* Self-Compassion Scale, *WHO-5* Five-item World Health Organization Well-Being Index

### Primary outcomes, feasibility and acceptability

#### Feasibility

Feasibility outcomes included attendance and dropout rates. After completing the courses, a course assistant with lived experience collected a de-identified list of attendees. Attendance was calculated based on the number of group sessions completed. Dropout was defined as any instance in which a participant did not complete the program [53]. Dropout rates were collected at the end of the program.

#### Acceptability

Acceptability was assessed after competition of the MSC program using the four-item Client Satisfaction Questionnaire (CSQ-4). In this study, the CSQ-4 [54], was used to measure the extent to which the program met the needs of the participants. The CSQ-4 assessed whether the received services helped the participants deal more effectively with their problems, how satisfied they were with the service, and whether they would return if they needed help again [55]. All items were measured using a four-point verbal anchor without a neutral position, ranging from “quite dissatisfied” to “very satisfied.” Each item was scored from 1 to 4, which resulted in a range of 4 to 16, where a higher score indicated greater satisfaction. In the present study, we used the validated Norwegian CSQ-4 version [55, 56].

### Secondary outcomes and preliminary effectiveness

To explore the preliminary effectiveness of the program, we assessed changes in self-compassion [57] and psychological well-being [58] from T1 to T2. These two outcomes were considered to be indicators of potential clinical impact but were not powered for statistical significance due to the small sample size.

#### Self-compassion

Self-compassion was measured using the Self-Compassion Scale (SCS), a 26-questions self-report scale that assesses a person’s self-compassion [57]. The SCS questions were answered based on the overarching question, “How do I typically treat myself when I’m having a hard time.” The SCS questionnaire is the most common instrument to measure self-reported self-compassion [7]. All SCS items were measured on a five-point scale, with 1 indicating that the person “almost never” thinks and feels as described in the statement or question and 5 indicating that the person “almost always” has such thoughts or feelings. The SCS form has questions related to the subscales of self-kindness, self-condemnation, common humanity, isolation, mindfulness, and over-identification (the latter indicates that a person’s identification with others is so strong that it inhibits their sense of self and independence). A score of 1 to 2.5 indicates low self-compassion, 2.5 to 3.5 indicates moderate self-compassion, and scores of 3.5 to 5.0 indicates high self-compassion.

#### Psychological well-being

Psychological well-being was assessed using the Five-item World Health Organization Well-Being Index (WHO-5). The WHO-5 includes five questions that measure the current experience of mental well-being based on one’s experience over the past two weeks. The measuring scale was first developed in 1998 at the WHO regional office in Europe as part of a project on instruments measuring well-being in primary care [58] and has been translated into Norwegian [59]. Each question is answered with six response alternatives, from zero, indicating “never,” to five, indicating “constantly.” The raw score is the sum of the five responses, from 0 (worst possible) to 25 (best possible mental well-being), multiplied by four, to obtain a standardized percentage score, varying between 0 (worst possible) and 100 (best possible mental well-being). If the raw score is below 13 or the person has answered 0 or 1 to any of the questions, further assessment for depression should be initiated. To detect change with the measuring instrument, Topp et al. (2015) considered a change of 10% to be significant, using a standardized percentage score [58]. In the present study, we used the validated Norwegian of the WHO-5 [59, 60].

### Description of the program

Two 8-week online MSC courses were delivered between January and December 2022. The MSC program was developed to strengthen self-compassion and is presented as a course rather than group therapy, spanning eight weeks and occurring once a week, including a half-day silent retreat [18]. The course was offered in groups via Zoom and it alternated between guided meditation and reflection, psychoeducation, experiential exercises, and group discussion. In addition, daily meditations were recommended as home tasks [61] to help participants cultivate self-compassion by acquiring tools they could incorporate into their daily routines.

The course introduces informal and formal self-compassion exercises, including exercises applicable in daily life (informal) and formal exercises, such as sitting meditation. The course also introduces mindfulness, which has a secondary emphasis. The course strongly emphasizes learning “loving-kindness” meditation, which involves developing a friendly and supportive attitude toward oneself in challenging everyday situations, rather than condemnation and punishment. Table 2 outlines the course structure and themes.

**Table 2:** Content of the course on mindful self-compassion

Sessions	Content
1	Introduction to MSC
2	Practicing mindfulness
3	Practicing kindness
4	Discovering one’s compassionate voice
5	Living deeply
6	Retreat
7	Addressing difficult emotions
8	Exploring challenging relationships
9	Embracing life

*MSC* Mindful Self-Compassion

The course content did not specifically cater to individuals with EDs. However, the theory and exercises were frequently linked to the body, food, and emotions to be relevant for this group. This was achieved by incorporating examples from daily life where food is typically used as a regulator and offering exercises that provided an alternative approach. The “silent retreat” (also digital) was an opportunity to renew and deepen the lessons learned in a quiet environment. This approach aimed to nurture a personal meditation practice and involved alternating between sitting, walking, yoga-based exercises, and group discussions.

To ensure fidelity, monitoring procedures were conducted weekly during the delivery of the program. In addition, the course instructor, trained in MSC (with the program’s founders), ensured a faithful reproduction of the original curriculum, providing detailed guidance on session content, exercises, and delivery methods. The course instructor also received supervision from the program developers, further supporting fidelity and adherence to the program.

### Statistical analyses and sample size

The missing data for all variables were below the recommended cutoff of 5% [6], leading to no imputation. In all analyses, missing values were excluded pairwise. The R (v. 4.4.1) software was employed in all analyses. Regarding the primary outcomes, dropout and attendance rates were calculated from the attendance registration and were reproduced as percentages. Acceptability of the program, measured using the CSQ-4, was calculated by calculating the mean (*M*) and standard deviation (*SD*), as well as the proportion of satisfaction, for each item [53].

For the secondary outcomes on the SCS and WHO-5, the *M*, confidence interval (CI) and *SD* were calculated for the subscales and total scores. The data distribution from WHO-5 was tested for normality using the Shapiro–Wilk test to determine whether the results were statistically significant. Although most data were normally distributed, the Wilcoxon–Mann–Whitney test was employed as an alternative to the most commonly used parametric *t*-test. Sensitivity analyses were not performed, given the exploratory nature of this pilot study. The significance level was set to 0.05. An average change estimate was calculated, and the associated effect size (Cohen’s *d*), to compare the data from T1 to the data from T2, detecting changes between the measurement points in the positive or negative direction. We calculated Cohen’s *d* [62] along with the corresponding 95% CIs*.* The 95% CIs for Cohen’s *d* were calculated based on the standard error, using an established approximation method for dependent samples. A Cohen’s *d* of 0.2 was considered a small effect size, 0.5 a medium effect size, and 0.8 a large effect size [62].

The sample size in this study was determined based on practical considerations related to conducting a pilot study, primarily driven by feasibility considerations, such as dropout and attendance rates, as well as the number of available participants during the data collection period. The number of participants was limited by the size of the target population during the recruitment period and by resource constraints within the study timeframe. Therefore, no formal power calculation was conducted.

## Results

### Participants

**Fig. 1:**
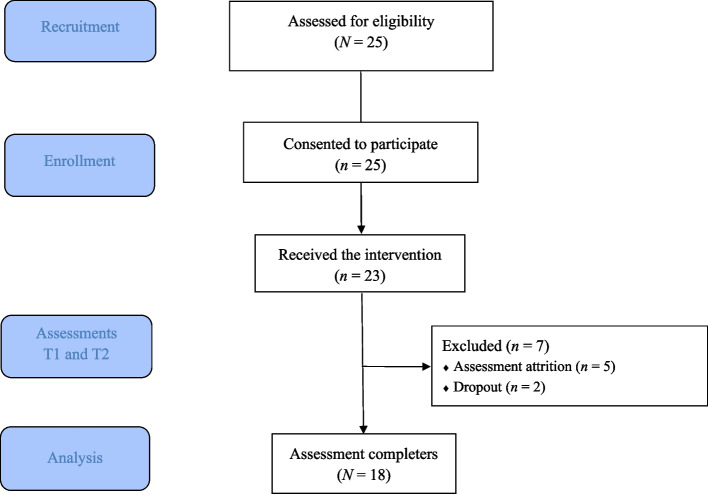
Study flowchart of patient progression through the study

The study flow is presented in Fig. 1. Twenty-five adults contacted the user organization to participate in the MSC, 12 in the spring of 2022 and 13 in the autumn of 2022. While 25 participants enrolled, 23 completed the program, and 18 (78%) completed both pre- and post-program measures.

Table 3 presents the sociodemographic characteristics. Eight (44%) participants self-reported an ED diagnosis: six with a binge ED, one with bulimia nervosa, and one with anorexia nervosa. The remaining 10 (56%) participants did not report a diagnosed ED at either survey time, but reported experiencing challenges related to food, body, and weight to varying degrees.

**Table 3:** Sociodemographic characteristics

Variable	Category	*n*	%
Sex	Woman	17	94%
	Man	1	6%
Age	< 30 years	2	11%
	30 to 39 years	2	11%
	40 to 49 years	2	11%
	50 to 59 years	10	56%
	60 years and older	2	11%
Highest education	Less than high school	0	0%
	High school	2	11%
	Bachelor’s degree or lower	8	44%
	Master’s degree or higher	8	44%
Marital status	Single	5	28%
	Partner/married	9	50%
	Separated/divorced	4	22%
	Widow/Widower	0	0%
Self-reported Eds	Yes	8	44%
	No	10	56%
Resident status	Living alone	7	39%
	Living with parent(s)	0	0%
	Living with child/children	2	11%
	Living with partner/spouse	8	44%
	Other	1	6%
Current main activity	Student	0	0%
	Paid job/self-employed	10	56%
	On sick leave	3	17%
	Laid off	0	0%
	Other	5	28%

*EDs* Eating disorders

### Primary outcomes: feasibility and acceptability

#### Feasibility

Feasibility outcomes included dropout and attendance rates. Two participants (8%) dropped out for personal reasons. These participants dropped out after the third and fifth group sessions, respectively.

Attendance rates were calculated based on the number of group sessions completed. The attendance rate was good, with 35% of the participants achieving 100% attendance, 39% completing seven of the eight group sessions (87.5% attendance), and 26% of the participants achieving 75% attendance.

#### Acceptability

Acceptability was assessed post-program using the CSQ-4. Results indicated that the participants were very satisfied with the online peer-led version of the MSC, with a total mean score of 14.4 (*SD* = 1.42, 95% CI [13.7, 15.1]) out of a maximum of 16 points. Sixteen participants (88.9%) reported being very satisfied with the services they received. In addition, 72.2% indicated that they would definitely use the program again (86.25%), and 11 participants (61.1%) reported that the program “helped a great deal” to cope with their problems. Most participants reported that their needs were met (55.6% reported “most of their needs” and 44.4% reported “almost all of the needs” were met).

### Secondary outcomes: preliminary effectiveness

We assessed the preliminary effectiveness of the program by measuring changes in self-compassion [57] and psychological well-being [58].

#### Self-compassion

As indicated in Table 4, the MSC program exhibited a moderate-to-large effect on the participants’ self-compassion (Cohen’s *d* = 0.68). From T1 to T2, the mean self-compassion score increased significantly from 2.50 (*SD* = 0.77) to 3.04 (*SD* = 0.83, *p* = 0.010).

**Table 4:** Score changes over time in self-compassion

**SCS scores**		**Time 1**	**Time 2**	**Paired *t*-test**			**Cohen’s *d***	
	***N***	**M (*SD*)**	**M (*SD*)**	**Diff**	***t***	***p***	***d***	**(95% CI)**
Self-Kindness	18	2.31 (0.79)	2.91 (0.88)	−0.60	−2.85	0.011	0.67	(0.60, 1.18)
Self-Judgement	18	2.28 (0.71)	2.74 (1.07)	−0.47	−1.83	0.085	0.43	(−0.05, 0.91)
Common Humanity	18	2.96 (1.00)	3.29 (0.90)	−0.33	−1.66	0.116	0.39	(−0.09, 0.87)
Isolation	18	2.56 (1.05)	3.06 (1.01)	−0.50	−2.33	0.033	0.55	(0.05, 1.05)
Mindfulness	18	2.67 (1.03)	3.26 (0.76)	−0.60	−2.80	0.012	0.66	(0.15, 1.17)
Over-Identification	18	2.32 (0.92)	3.07 (0.98)	−0.75	−3.10	0.006	0.73	(0.21, 1.25)
Total	18	2.50 (0.77)	3.04 (0.83)	−0.54	−2.90	0.010	0.68	(0.17, 1.19)

*T1* before the program, *T2* after the program, *SCS* Self-Compassion Scale, *SD* standard deviation, *CI* confidence interval, *d* effect size (Cohen’s *d*). The 95% CIs for Cohen’s *d* were calculated based on the standard error, using an established approximation method for dependent samples

#### Psychological well-being

Table 5 presents the changes over time in psychological well-being outcomes. A conditional *t*-test was employed because the *p*-value for the Wilcoxon signed-rank test was just above the significance level. Based on this, the MSC had a moderate effect on the perception of psychological well-being for participants (Cohen’s *d* = 0.52); the *t*-test displays a significant change (*p* < 0.05 = 0.041). The mean value for the raw score increased from 8.5 (*SD* = 4.36) at T1 to 10.70 (*SD* = 4.71) at T2. Converting these scores to standardized percentage scores [58], it yielded 34.0 for T1 and 42.8 for T2, considered clinically significant.

**Table 5:** Score changes over time in WHO-5 Well-Being Index

**WHO-5 scores**		**Time 1**	**Time 2**	**Paired *t*-test**			**Cohen’s *d***	
	***n***	**M (*SD*)**	**M (*SD*)**	**Diff**	***t***	***p***	***d***	**(95% CI)**
Item 1	18	1.94 (1.11)	2.28 (1.27)	−0.33	−1.19	0.250	0.28	(−0.19, 0.75)
Item 2	18	1.89 (1.13)	2.39 (1.14)	−0.50	−1.76	0.095	0.41	(−0.07, 0.89)
Item 3	18	1.28 (1.23)	1.61 (1.29)	−0.33	−1.37	0.187	0.32	(−0.15, 0.79)
Item 4	18	1.33 (1.19)	1.89 (1.23)	−0.55	−2.05	0.055	0.48	(−0.01, 0.97)
Item 5	18	2.06 (1.26)	2.56 (1.20)	−0.50	−1.53	0.143	0.36	(−0.12, 0.84)
Total	18	8.50 (4.36)	10.7 (4.71)	−2.22	−2.21	0.041	0.52	(0.03, 1.01)

*T1* before the program, *T2* after the program, *WHO-5* World Health Organization Well-Being Index, *SD* standard deviation, *CI* confidence interval, *d* effect size (Cohen’s *d*). The 95% CIs for Cohen’s *d* were calculated based on the standard error, using an established approximation method for dependent samples

## Discussion

This study aimed to evaluate the feasibility, acceptability and preliminary effectiveness of an online peer-led MSC program for adults with EDs symptoms. A total of 23 adults completed the program. The findings suggest that this online program was both feasible and acceptable.

Regarding feasibility outcomes, the MSC group program demonstrated strong feasibility with a high program completion rate and. Only two participants dropped out. This finding aligns with previous studies reporting excellent retention rates in similar MSC programs among other populations [18, 21, 22, 42]. Furthermore, this finding aligns with a systematic review by Fortuna et al. (2020), which indicated the feasibility of web-based peer-led interventions for people with mental health problems [27]. However, the evidence related to digital programs remains limited in the context of EDs [38]. Feasibility was also assessed through attendance rates, calculated based on the number of group sessions completed, with most participants completing the program. This result aligns with the recent study of an online video-based MSC with peer-led support [21], which suggests that an online delivery format may be a promising approach for engaging individuals with ED symptoms in self-compassion practices.

We assessed acceptability through client satisfaction ratings, as suggested when measuring acceptability with new health programs [63]. The study findings suggest that the participants were very satisfied with the online peer-led MSC program, due to high scores on individual questions, with most participants reporting being very satisfied with the services they received. This finding corresponds with previous studies suggesting that programs including lived experience and peer support are well-received by adults with mental health challenges [64, 65].

Additionally, two-thirds of the participants indicated that they would use the program again. Notably, 23 of the 25 participants completed the courses, which indicated a high level of acceptance of the program. These preliminary results support previous research that suggests that peer support models are well-received by individuals with EDs [35]. However, measuring the acceptability of peer-led programs warrants further research to identify the specific program components that contribute to participant satisfaction in the program.

Regarding the secondary outcomes, the study findings revealed that the average self-compassion score improved pre- to post-program. Our preliminary results align with previous studies suggesting that MSC programs may be beneficial as additional approaches for women with EDs [9, 18, 22]. These studies demonstrated that MSC significantly improved self-compassion in the general population [18], and in individuals with chronic pain [22].

However, even though our preliminary evidence suggested that MSC had a moderate effect on the psychological well-being of people with ED symptoms, the average raw score, which increased from 8.50 at T1 to 10.7 at T2, was still low. According to Topp et al. (2015), this low score indicates that the participants should be assessed for depression [58]. Nevertheless, the current results suggest that the MSC program improved the psychological well-being of those with ED symptoms, aligned with previous studies [21, 22]. However, the research regarding online group-based peer-led programs is sparse, so our preliminary findings should be interpreted cautiously.

### Strengths and limitations

This study had several strengths. The design applied a PAR approach that actively involved adults with lived experience of EDs as co-facilitators and co-creators, as lived experience experts delivered and managed the program and collected the data. This co-creating approach aligns with the current policy landscape, which advocates for incorporating lived experience in the co-production and co-delivery of EDs services [26], as well as a growing recognition of the value of lived experience in improving mental health services [27]. Additionally, the peer-led design emphasized mutual support and experiential knowledge. Through close collaboration with a user organization, during the planning, delivery, and evaluation phases, this study increased its ecological validity by being grounded in real-world experiences. Furthermore, the feasibility of delivering a structured MSC in an online format, specifically via Zoom, was successfully demonstrated, allowing people from different regions of Norway to participate in the program, potentially including individuals who might not have access to traditional in-person peer-led services. Furthermore, the collection of both quantitative data on feasibility and satisfaction strengthened the foundation for future research and practical application.

However, these findings were potentially limited by the use of self-report questionnaires to explore the preliminary effectiveness of the program. This self-report method could have introduced a response bias, where the participants may have underreported symptoms or overreported satisfaction scores. However, the data collection process was anonymous to reduce the potential risk of social desirability bias.

Our findings were potentially limited in terms of their generalizability, as they were specific to a Nordic context. The program was conducted in Norway, a country with a very well-funded public health system [66]. In addition, residents of Norway have a relatively high level of access and participation in online systems, which results in high digital literacy. Furthermore, there is good precedent and openness to peer-led interventions. Therefore, the cultural background and other contextual societal factors could have influenced openness to self-compassion program. In contrast, attitudes toward peer-led interventions and self-compassion could have varied across cultures, which potentially limited the generalizability of these results.

The sample group was relatively homogeneous in terms of gender and educational background, which might restrict the relevance of the findings to more diverse EDs populations. This heterogeneity and the sample size limitations made it difficult to conduct specific subgroup analyses, which potentially masked the differential effects of the program. Lastly, while the study assessed preliminary effectiveness and change over time, it lacked a control group, and did not collect follow-up data. Both of these factors limited the ability to understand and explore the long-term effects or directly attribute behavioral changes to the program.

### Implications and future directions

Although no harm was reported in the current study, self-compassion programs, particularly in group settings, carry potential risks [67, 68]. Engaging in self-reflective practices can evoke emotional distress, especially in individuals with a history of self-criticism. This experience is common among those with EDs [16]. A potential risk associated with participating in online group sessions is the discomfort experienced by participants when confronted by difficult emotions (e.g. shame or discomfort in sharing personal experiences) [68, 69].

Therefore, future implementations and research must recognize these inherent challenges. Given the preliminary evidence of both benefits and possible risks [70], future research can monitor potential harm and incorporate feedback from patients and peer facilitators to refine the MSC program. This feedback can include systematically collecting feedback from participants, especially regarding their emotional experiences and perceived risks. Embedding these feedback mechanisms can support ongoing refinement while ensuring psychological safety. Moreover, clinical trials in more homogenous groups are needed to further investigate whether the online group-based peer-led MSC program is effective. Further research can assess ED symptoms pre- and post-program, with follow-up studies to evaluate the short- and long-term effects.

Furthermore, future studies can evaluate the experiences of the course leader and the user organization providing the program. Qualitative research is recommended to explore the impact on the course instructor with the lived experience and whether working with people with ED symptoms poses challenges or impacts their lives and recoveries. As research into peer-led online group programs for people with EDs is still emerging, future qualitative studies can provide a better understanding of the need for supervision and training for group leaders to guide the further implementation of these programs. Furthermore, qualitative studies can offer valuable insights into what users find beneficial, the barriers they face and what facilitates their participation [50].

## Conclusions

This study aimed to evaluate the feasibility, acceptability, and preliminary effectiveness of an online peer-led group MSC program for adults experiencing ED challenges. Our results indicate that the program shows promise for adults with ED symptoms since the program was both feasible and acceptable to this group. With only two participants dropping out and a high attendance rate, the program demonstrated strong engagement. The participants reported strong satisfaction with the program and significant improvements in self-compassion and psychological well-being scores.

As this study is the first to evaluate an online group-based peer-led program in this context, these preliminary findings should be interpreted with caution. Further studies are needed to replicate these results. Additionally, future research could explore specific program components that contribute to participant satisfaction, to gain a deeper understanding of what users find beneficial and which factors facilitate their participation. However, these findings suggest that peer-led online MSC programs may offer an accessible, community-rooted, and person-centered addition to ED services, particularly for individuals underserved by traditional models.

## Data Availability

The dataset that supports the findings of the current study is not publicly available due to ethical and legal reasons.

## Abbreviations

CI: Confidence interval
CSQ-4: Client Satisfaction Questionnaire, 4-item version
*D*: Effect size, Cohen’s *d*
ED: Eating disorder
MSC: Mindful Self-Compassion
*M*: Mean
Mdn: Median
REK: Regionale komiteer for medisinsk og helsefaglig forskningsetikk. Translated: Regional Ethical Committee
*SD*: Standard Deviation
SCS: Self-Compassion Scale
T1: Time 1, before the program
T2: Time 2, after the program
WHO-5: The Five-item World Health Organization Well-Being Index
